# Gliclazide impurity F: *N*-[(perhydro­cyclo­penta­[*c*]pyrrol-2-yl)amino­carbon­yl]-*o*-toluene­sulfonamide

**DOI:** 10.1107/S1600536811054985

**Published:** 2012-01-18

**Authors:** Di Wu, Xueyuan Wang, Dongying Pang, Wei Su, Yan Sun

**Affiliations:** a Department of Chemistry, School of Science, Tianjin University, Tianjin 300072, People’s Republic of China; b TianJin Centralpharm Limited Company, Tianjin 300072, People’s Republic of China; c High Pressure Adsorption Laboratory, School of Chemical Engineering and Technology, Tianjin University, Tianjin 300072, People’s Republic of China

## Abstract

The title compound, C_15_H_21_N_3_O_3_S, is known to be an impurity of gliclazide [systematic name: *N*-(hexa­hydro-1*H*-cyclopenta[*c*]pyrrol-2-ylcarbamo­yl)-4-methyl­benzene­sulfonamide], a sul­fonyl­urea anti­diabetic drug. Gliclazide has a *p*-tolyl group substituting the sulfonamide functionality, while the title mol­ecule contains an *o*-tolyl group. Both five-membered fused rings adopt envelope conformations. In the crystal, N—H⋯O hydrogen bonds are formed between HN(C=O)NH groups, building centrosymmetric dimers. These dimers are further linked through N—H⋯O(sulfon­yl) contacts, forming chains in [100].

## Related literature

For general background to gliclazide and the impurities of gliclazide, see: Lebovitz & Feinglos (1983 ▶). For the crystal structure of gliclazide, see: Parvez *et al.* (1999 ▶); Winters *et al.* (1994 ▶).
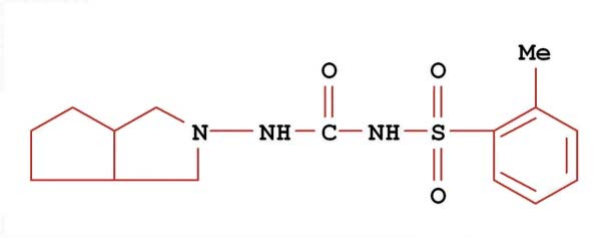


## Experimental

### 

#### Crystal data

C_15_H_21_N_3_O_3_S
*M* *_r_* = 323.41
Monoclinic, 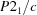

*a* = 10.891 (7) Å
*b* = 11.226 (7) Å
*c* = 13.477 (9) Å
β = 95.509 (9)°
*V* = 1640.2 (18) Å^3^
*Z* = 4
Mo *K*α radiation
μ = 0.21 mm^−1^
*T* = 113 K
0.20 × 0.18 × 0.10 mm

#### Data collection

Rigaku Saturn724 CCD diffractometer
Absorption correction: multi-scan (*CrystalClear*; Rigaku/MSC, 2002 ▶) *T*_min_ = 0.959, *T*_max_ = 0.979
16805 measured reflections
3904 independent reflections
3461 reflections with *I* > 2σ(*I*)
*R*_int_ = 0.070

#### Refinement

*R*[*F* ^2^ > 2σ(*F* ^2^)] = 0.044
*wR*(*F* ^2^) = 0.113
*S* = 1.04
3904 reflections
208 parameters
3 restraints
H atoms treated by a mixture of independent and constrained refinement
Δρ_max_ = 0.30 e Å^−3^
Δρ_min_ = −0.51 e Å^−3^

### 

Data collection: *CrystalClear* (Rigaku/MSC, 2002 ▶); cell refinement: *CrystalClear*; data reduction: *CrystalClear*; program(s) used to solve structure: *SHELXS97* (Sheldrick, 2008 ▶); program(s) used to refine structure: *SHELXL97* (Sheldrick, 2008 ▶); molecular graphics: *SHELXTL* (Sheldrick, 2008 ▶); software used to prepare material for publication: *CrystalStructure* (Rigaku/MSC, 2006 ▶).

## Supplementary Material

Crystal structure: contains datablock(s) I, global. DOI: 10.1107/S1600536811054985/bh2389sup1.cif

Structure factors: contains datablock(s) I. DOI: 10.1107/S1600536811054985/bh2389Isup2.hkl

Supplementary material file. DOI: 10.1107/S1600536811054985/bh2389Isup3.cml

Additional supplementary materials:  crystallographic information; 3D view; checkCIF report

## Figures and Tables

**Table 1:** Hydrogen-bond geometry (Å, °)

*D*—H⋯*A*	*D*—H	H⋯*A*	*D*⋯*A*	*D*—H⋯*A*
N2—H2⋯O3^i^	0.90 (1)	1.92 (1)	2.820 (2)	177 (2)
N1—H1⋯O2^ii^	0.89 (1)	2.23 (1)	3.077 (2)	158 (2)

Symmetry codes: (i) 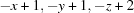; (ii) 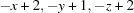.

**Fig. 1.**
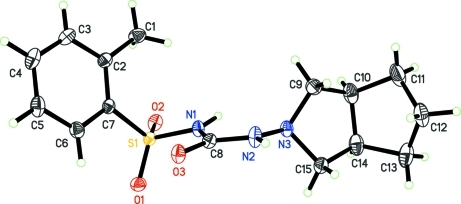
The title molecule with displacement ellipsoids for non-H atoms at the 50% probability level.

## supplementary crystallographic information

### Comment

Gliclazide Impurity F is one of the gliclazide impurities, as described in the *European Pharmacopoeia*. Gliclazide is a second-generation sulfonylurea oral hypoglycemic agent, which can reduce blood sugar and improve blood clotting function (Lebovitz & Feinglos, 1983). It not only can improve the metabolism of diabetic patients, but also improve or delay the incidence of vascular complications of diabetes.

The reason for the existence of this impurity is mainly due to the generation of *ortho*-isomeride during the production process of raw material, 4-methylphenylsulfonylurea or ethyl-[(4-methylphenyl)sulfonyl]carbamate. In the liquid chromatography separation experiments, the *ortho* gliclazide derivative has frequently been used as working sample. In this paper, we report the crystal structure of this compound.

The molecular structure is shown in Fig. 1. Molecular dimensions are within the normal ranges. The similar corresponding bond distances and angles have been reported in structure of gliclazide (Parvez *et al.*, 1999; Winters *et al.*, 1994). The methyl position of toluenesulfonyl moiety is the important difference between the two structures. The mean bond distances in the *o*-toluenesulfonyl moiety are C—C_aromatic_ = 1.507 (2), S=O = 1.4354 (14), C*sp*^2^-C*sp*^2^=1.394 (3) and S—C*sp*^2^ = 1.7690 (18) Å, the aromatic ring being essentially planar. The mean values of the bond distances in the perhydrocyclopenta[*c*]pyrrole moiety in the title compound are C*sp*^3^-C*sp*^3^ =1.536 (3) and C*sp*^3^-N=1.473 (2) Å. The pyrrole (N3, C9, C10, C14, C15) ring and the fused five-membered cyclopentane (C10···C14) adopt N3- and C12- envelope conformations, respectively, with N3 0.624 and C12 0.611Å out of the planes of the remaining atoms of the corresponding rings.

The molecules are linked into dimers by intermolecular hydrogen bonds involving amino H-atoms. Intermolecular contacts between symmetry-related dimers form chains in the [100] direction.

### Experimental

A 1000 ml reactor fitted with an electric heater in the bottom, a mechanical stirrer, a thermometer and a condenser was loaded with urea (100 g) and sodium hydroxide (5 g). Slow heating and addition of *o*-toluenesulfonamide (48 g) after the reactants changed the mixture to the molten state. When the reaction was completed after 6 h, water and hydrochloric acid were added until pH = 7. After filtering, draining and vacuum drying, the *o*-toluene sulfonylurea was obtained (yield 90%). The *o*-toluene sulfonylurea was added to the equal amount of azolidine hydrochloride in acetonitrile under reflux for 6 h. Then, the desired products were obtained after cooling down, and filtered (yield 86%). Finally, the products were recrystallized from methanol.

### Refinement

H atoms were positioned geometrically, with C—H bond lengths fixed to 0.95 (aromatic CH), 0.98 (methyl CH_3_), 0.99 (methylene CH_2_) or 1.00 Å (methine CH), and constrained to ride on their parent atoms. H atoms bonded to N1 and N2 were refined freely, with N—H bond lengths restrained to 0.90 (1) Å. For all H atoms, isotropic displacement parameters were calculated as *U*_iso_(H) = *xU*_eq_(carrier atom), where *x* = 1.2 or 1.5.

### Crystal data

**Table:**

C_15_H_21_N_3_O_3_S	*F*(000) = 688
*M**_r_* = 323.41	*D*_x_ = 1.310 Mg m^−^^3^
Monoclinic, *P*2_1_/*c*	Mo *K*α radiation, λ = 0.71073 Å
Hall symbol: -P 2ybc	Cell parameters from 5900 reflections
*a* = 10.891 (7) Å	θ = 1.5–27.9°
*b* = 11.226 (7) Å	µ = 0.21 mm^−^^1^
*c* = 13.477 (9) Å	*T* = 113 K
β = 95.509 (9)°	Prism, colourless
*V* = 1640.2 (18) Å^3^	0.20 × 0.18 × 0.10 mm
*Z* = 4	

### Data collection

**Table:**

Rigaku Saturn724 CCD diffractometer	3904 independent reflections
Radiation source: rotating anode	3461 reflections with *I* > 2σ(*I*)
multilayer	*R*_int_ = 0.070
Detector resolution: 14.22 pixels mm^-1^	θ_max_ = 27.9°, θ_min_ = 1.9°
ω and φ scans	*h* = −14→14
Absorption correction: multi-scan (*CrystalClear*; Rigaku/MSC, 2002)	*k* = −14→13
*T*_min_ = 0.959, *T*_max_ = 0.979	*l* = −17→17
16805 measured reflections	

### Refinement

**Table:**

Refinement on *F*^2^	Primary atom site location: structure-invariant direct methods
Least-squares matrix: full	Secondary atom site location: difference Fourier map
*R*[*F*^2^ > 2σ(*F*^2^)] = 0.044	Hydrogen site location: inferred from neighbouring sites
*wR*(*F*^2^) = 0.113	H atoms treated by a mixture of independent and constrained refinement
*S* = 1.04	*w* = 1/[σ^2^(*F*_o_^2^) + (0.0562*P*)^2^ + 0.7703*P*] where *P* = (*F*_o_^2^ + 2*F*_c_^2^)/3
3904 reflections	(Δ/σ)_max_ = 0.002
208 parameters	Δρ_max_ = 0.30 e Å^−^^3^
3 restraints	Δρ_min_ = −0.51 e Å^−^^3^
0 constraints	

### Fractional atomic coordinates and isotropic or equivalent isotropic displacement parameters (Å^2^)

**Table:**

	*x*	*y*	*z*	*U*_iso_*/*U*_eq_	
S1	0.88574 (3)	0.63900 (3)	1.09521 (2)	0.01593 (11)	
O1	0.83163 (10)	0.75502 (10)	1.08708 (8)	0.0226 (2)	
O2	1.01217 (9)	0.62502 (10)	1.07406 (8)	0.0209 (2)	
O3	0.62260 (9)	0.57766 (10)	1.07603 (8)	0.0205 (2)	
N1	0.80984 (11)	0.54879 (12)	1.01545 (9)	0.0174 (3)	
N2	0.63131 (12)	0.47495 (13)	0.93204 (9)	0.0222 (3)	
N3	0.69959 (11)	0.44335 (12)	0.85221 (9)	0.0179 (3)	
C1	1.01300 (15)	0.40745 (16)	1.19750 (12)	0.0251 (3)	
H1A	0.9694	0.3882	1.1324	0.038*	
H1B	1.0329	0.3337	1.2345	0.038*	
H1C	1.0893	0.4503	1.1879	0.038*	
C2	0.93199 (14)	0.48450 (14)	1.25551 (11)	0.0190 (3)	
C3	0.91819 (16)	0.45650 (16)	1.35526 (12)	0.0250 (3)	
H3	0.9612	0.3899	1.3850	0.030*	
C4	0.84335 (16)	0.52348 (17)	1.41160 (12)	0.0288 (4)	
H4	0.8377	0.5035	1.4795	0.035*	
C5	0.77680 (16)	0.61910 (17)	1.36998 (12)	0.0266 (4)	
H5	0.7238	0.6632	1.4083	0.032*	
C6	0.78836 (15)	0.64984 (15)	1.27155 (11)	0.0211 (3)	
H6	0.7428	0.7150	1.2420	0.025*	
C7	0.86715 (13)	0.58463 (14)	1.21606 (10)	0.0165 (3)	
C8	0.68259 (13)	0.53668 (14)	1.01056 (10)	0.0169 (3)	
C9	0.70155 (15)	0.31371 (15)	0.83580 (12)	0.0235 (3)	
H9A	0.6184	0.2788	0.8382	0.028*	
H9B	0.7598	0.2739	0.8862	0.028*	
C10	0.74462 (16)	0.30259 (17)	0.73176 (13)	0.0289 (4)	
H10	0.8364	0.2946	0.7354	0.035*	
C11	0.68091 (18)	0.20163 (19)	0.66902 (16)	0.0389 (5)	
H11A	0.7340	0.1726	0.6185	0.047*	
H11B	0.6607	0.1341	0.7118	0.047*	
C12	0.56419 (18)	0.25961 (19)	0.61967 (14)	0.0358 (4)	
H12A	0.5304	0.2138	0.5605	0.043*	
H12B	0.5003	0.2661	0.6669	0.043*	
C13	0.6079 (2)	0.3821 (2)	0.58998 (13)	0.0386 (5)	
H13A	0.5380	0.4386	0.5806	0.046*	
H13B	0.6484	0.3777	0.5274	0.046*	
C14	0.70093 (17)	0.42092 (17)	0.67816 (12)	0.0294 (4)	
H14	0.7720	0.4652	0.6541	0.035*	
C15	0.64125 (16)	0.49198 (16)	0.75762 (11)	0.0238 (3)	
H15A	0.6586	0.5782	0.7520	0.029*	
H15B	0.5508	0.4797	0.7519	0.029*	
H1	0.8539 (17)	0.5085 (18)	0.9741 (13)	0.038 (6)*	
H2	0.5498 (9)	0.4609 (18)	0.9291 (15)	0.031 (5)*	

### Atomic displacement parameters (Å^2^)

**Table:**

	*U*^11^	*U*^22^	*U*^33^	*U*^12^	*U*^13^	*U*^23^
S1	0.01388 (18)	0.0184 (2)	0.01533 (18)	−0.00225 (13)	0.00034 (13)	0.00038 (12)
O1	0.0254 (6)	0.0186 (6)	0.0233 (5)	−0.0009 (5)	−0.0007 (4)	0.0021 (4)
O2	0.0125 (5)	0.0291 (6)	0.0212 (5)	−0.0041 (4)	0.0017 (4)	0.0003 (4)
O3	0.0147 (5)	0.0292 (6)	0.0180 (5)	0.0003 (4)	0.0028 (4)	−0.0054 (4)
N1	0.0125 (6)	0.0234 (7)	0.0162 (6)	−0.0016 (5)	0.0014 (4)	−0.0045 (5)
N2	0.0130 (6)	0.0359 (8)	0.0181 (6)	−0.0031 (5)	0.0037 (5)	−0.0082 (5)
N3	0.0158 (6)	0.0229 (7)	0.0155 (6)	−0.0003 (5)	0.0036 (5)	−0.0040 (5)
C1	0.0235 (8)	0.0263 (9)	0.0257 (8)	0.0063 (7)	0.0045 (6)	0.0044 (6)
C2	0.0158 (7)	0.0215 (8)	0.0192 (7)	−0.0010 (6)	−0.0007 (5)	0.0007 (6)
C3	0.0256 (8)	0.0292 (9)	0.0199 (7)	0.0010 (7)	−0.0005 (6)	0.0062 (6)
C4	0.0303 (9)	0.0399 (11)	0.0163 (7)	−0.0038 (8)	0.0029 (6)	0.0014 (7)
C5	0.0245 (8)	0.0366 (10)	0.0192 (7)	0.0001 (7)	0.0043 (6)	−0.0064 (7)
C6	0.0193 (7)	0.0225 (8)	0.0212 (7)	0.0002 (6)	0.0008 (6)	−0.0034 (6)
C7	0.0148 (6)	0.0188 (7)	0.0153 (6)	−0.0038 (6)	−0.0011 (5)	−0.0012 (5)
C8	0.0138 (6)	0.0204 (8)	0.0165 (6)	−0.0002 (6)	0.0018 (5)	0.0003 (5)
C9	0.0210 (7)	0.0224 (8)	0.0265 (8)	0.0013 (6)	−0.0010 (6)	−0.0019 (6)
C10	0.0202 (7)	0.0357 (10)	0.0308 (9)	0.0020 (7)	0.0027 (6)	−0.0136 (7)
C11	0.0335 (10)	0.0372 (11)	0.0447 (11)	0.0047 (8)	−0.0033 (8)	−0.0228 (9)
C12	0.0309 (9)	0.0407 (11)	0.0347 (9)	−0.0030 (8)	−0.0015 (7)	−0.0179 (8)
C13	0.0440 (11)	0.0524 (13)	0.0187 (8)	−0.0078 (10)	0.0001 (7)	−0.0064 (8)
C14	0.0323 (9)	0.0363 (10)	0.0204 (7)	−0.0092 (8)	0.0070 (7)	−0.0059 (7)
C15	0.0295 (8)	0.0234 (9)	0.0181 (7)	−0.0013 (7)	−0.0002 (6)	−0.0003 (6)

### Geometric parameters (Å, °)

**Table:**

S1—O1	1.4295 (14)	C5—H5	0.9500
S1—O2	1.4413 (14)	C6—C7	1.398 (2)
S1—N1	1.6413 (14)	C6—H6	0.9500
S1—C7	1.7690 (18)	C9—C10	1.526 (2)
O3—C8	1.2360 (18)	C9—H9A	0.9900
N1—C8	1.388 (2)	C9—H9B	0.9900
N1—H1	0.893 (7)	C10—C11	1.539 (2)
N2—C8	1.341 (2)	C10—C14	1.564 (3)
N2—N3	1.4107 (17)	C10—H10	1.0000
N2—H2	0.898 (9)	C11—C12	1.523 (3)
N3—C9	1.473 (2)	C11—H11A	0.9900
N3—C15	1.474 (2)	C11—H11B	0.9900
C1—C2	1.507 (2)	C12—C13	1.521 (3)
C1—H1A	0.9800	C12—H12A	0.9900
C1—H1B	0.9800	C12—H12B	0.9900
C1—H1C	0.9800	C13—C14	1.548 (3)
C2—C3	1.403 (2)	C13—H13A	0.9900
C2—C7	1.405 (2)	C13—H13B	0.9900
C3—C4	1.388 (2)	C14—C15	1.529 (2)
C3—H3	0.9500	C14—H14	1.0000
C4—C5	1.384 (3)	C15—H15A	0.9900
C4—H4	0.9500	C15—H15B	0.9900
C5—C6	1.388 (2)		
			
O1—S1—O2	118.60 (7)	N3—C9—C10	103.24 (13)
O1—S1—N1	109.51 (8)	N3—C9—H9A	111.1
O2—S1—N1	103.53 (7)	C10—C9—H9A	111.1
O1—S1—C7	107.59 (7)	N3—C9—H9B	111.1
O2—S1—C7	109.91 (7)	C10—C9—H9B	111.1
N1—S1—C7	107.17 (8)	H9A—C9—H9B	109.1
C8—N1—S1	121.96 (10)	C9—C10—C11	113.77 (16)
C8—N1—H1	121.0 (14)	C9—C10—C14	104.37 (13)
S1—N1—H1	117.1 (14)	C11—C10—C14	105.70 (15)
C8—N2—N3	121.44 (13)	C9—C10—H10	110.9
C8—N2—H2	117.4 (13)	C11—C10—H10	110.9
N3—N2—H2	120.9 (13)	C14—C10—H10	110.9
N2—N3—C9	112.29 (12)	C12—C11—C10	103.80 (16)
N2—N3—C15	110.58 (13)	C12—C11—H11A	111.0
C9—N3—C15	104.33 (12)	C10—C11—H11A	111.0
C2—C1—H1A	109.5	C12—C11—H11B	111.0
C2—C1—H1B	109.5	C10—C11—H11B	111.0
H1A—C1—H1B	109.5	H11A—C11—H11B	109.0
C2—C1—H1C	109.5	C13—C12—C11	103.40 (17)
H1A—C1—H1C	109.5	C13—C12—H12A	111.1
H1B—C1—H1C	109.5	C11—C12—H12A	111.1
C3—C2—C7	116.48 (14)	C13—C12—H12B	111.1
C3—C2—C1	119.38 (15)	C11—C12—H12B	111.1
C7—C2—C1	124.14 (14)	H12A—C12—H12B	109.0
C4—C3—C2	121.70 (16)	C12—C13—C14	104.54 (16)
C4—C3—H3	119.1	C12—C13—H13A	110.8
C2—C3—H3	119.1	C14—C13—H13A	110.8
C5—C4—C3	120.74 (15)	C12—C13—H13B	110.8
C5—C4—H4	119.6	C14—C13—H13B	110.8
C3—C4—H4	119.6	H13A—C13—H13B	108.9
C4—C5—C6	119.22 (15)	C15—C14—C13	113.18 (16)
C4—C5—H5	120.4	C15—C14—C10	104.51 (14)
C6—C5—H5	120.4	C13—C14—C10	105.24 (16)
C5—C6—C7	119.84 (16)	C15—C14—H14	111.2
C5—C6—H6	120.1	C13—C14—H14	111.2
C7—C6—H6	120.1	C10—C14—H14	111.2
C6—C7—C2	121.92 (14)	N3—C15—C14	103.62 (14)
C6—C7—S1	116.24 (12)	N3—C15—H15A	111.0
C2—C7—S1	121.79 (11)	C14—C15—H15A	111.0
O3—C8—N2	123.16 (14)	N3—C15—H15B	111.0
O3—C8—N1	121.58 (14)	C14—C15—H15B	111.0
N2—C8—N1	115.24 (13)	H15A—C15—H15B	109.0
			
O1—S1—N1—C8	−51.88 (14)	N3—N2—C8—O3	171.41 (14)
O2—S1—N1—C8	−179.30 (12)	N3—N2—C8—N1	−10.5 (2)
C7—S1—N1—C8	64.54 (14)	S1—N1—C8—O3	−12.1 (2)
C8—N2—N3—C9	122.19 (16)	S1—N1—C8—N2	169.83 (12)
C8—N2—N3—C15	−121.73 (16)	N2—N3—C9—C10	163.97 (12)
C7—C2—C3—C4	0.7 (2)	C15—N3—C9—C10	44.19 (15)
C1—C2—C3—C4	−179.07 (16)	N3—C9—C10—C11	−142.41 (15)
C2—C3—C4—C5	1.7 (3)	N3—C9—C10—C14	−27.70 (16)
C3—C4—C5—C6	−1.9 (3)	C9—C10—C11—C12	87.0 (2)
C4—C5—C6—C7	−0.3 (2)	C14—C10—C11—C12	−26.96 (19)
C5—C6—C7—C2	2.8 (2)	C10—C11—C12—C13	41.04 (19)
C5—C6—C7—S1	−174.57 (12)	C11—C12—C13—C14	−39.17 (19)
C3—C2—C7—C6	−2.9 (2)	C12—C13—C14—C15	−91.52 (19)
C1—C2—C7—C6	176.81 (15)	C12—C13—C14—C10	22.00 (19)
C3—C2—C7—S1	174.32 (12)	C9—C10—C14—C15	2.29 (17)
C1—C2—C7—S1	−5.9 (2)	C11—C10—C14—C15	122.56 (15)
O1—S1—C7—C6	10.97 (14)	C9—C10—C14—C13	−117.18 (15)
O2—S1—C7—C6	141.43 (12)	C11—C10—C14—C13	3.09 (18)
N1—S1—C7—C6	−106.71 (13)	N2—N3—C15—C14	−163.62 (13)
O1—S1—C7—C2	−166.44 (12)	C9—N3—C15—C14	−42.68 (16)
O2—S1—C7—C2	−35.98 (14)	C13—C14—C15—N3	137.91 (16)
N1—S1—C7—C2	75.88 (14)	C10—C14—C15—N3	23.94 (17)

### Hydrogen-bond geometry (Å, °)

**Table:**

*D*—H···*A*	*D*—H	H···*A*	*D*···*A*	*D*—H···*A*
N2—H2···O3^i^	0.90 (1)	1.92 (1)	2.820 (2)	177 (2)
N1—H1···O2^ii^	0.89 (1)	2.23 (1)	3.077 (2)	158 (2)

Symmetry codes: (i) −*x*+1, −*y*+1, −*z*+2; (ii) −*x*+2, −*y*+1, −*z*+2.
